# Phase 1 study of safety, tolerability, and efficacy of intradermal DNA vaccine ASP2390 in adults allergic to house dust mites

**DOI:** 10.1016/j.jacig.2025.100404

**Published:** 2025-01-07

**Authors:** Thomas Kayser, Ronald Smulders, Tomohiro Kusawake, Erik Wambre, Gurunadh R. Chichili, Mary B. Blauwet, Anna Spence, Melanie Patton, Rima Tabash, Hannah A. DeBerg, Sugandhika Khosa, Philipp Badorrek, Jens M. Hohlfeld, Brian C. Ferslew

**Affiliations:** a Fraunhofer Institute for Toxicology and Experimental Medicine, Hannover, Germany; b Astellas Pharma Global Development Inc, Northbrook, Ill; c Astellas Pharma Inc, Tokyo, Japan; d Benaroya Research Institute at Virginia Mason, Seattle, Wash; e Department of Respiratory Medicine, Hannover Medical School, Hannover, Germany; f German Center for Lung Research (DZL), Biomedical Research in Endstage and Obstructive Lung Disease Hannover BREATH, Hannover, Germany

**Keywords:** Allergen immunotherapy, allergy, ASP2390, clinical trial, house dust mite, immunologic response, LAMP-based DNA vaccine, phase 1, safety, tolerability

## Abstract

**Background:**

House dust mite (HDM) allergies are prevalent, yet current treatments like allergen avoidance, pharmacotherapy, and conventional allergen immunotherapy present limitations. The novel LAMP (lysosomal-associated membrane protein)-based DNA vaccine ASP2390 targets major HDM allergens, potentially shifting immune responses toward nonallergic pathways and minimizing the risk of atopy, with positive safety and efficacy signals in preclinical models.

**Objective:**

We evaluated the safety, tolerability, and efficacy of first-in-human intradermal ASP2390 in adults with HDM allergy.

**Methods:**

A randomized, double-blind, placebo-controlled phase 1 trial was conducted in adults with HDM-induced allergic rhinitis. Participants received either 1 mg or 4 mg of ASP2390 or placebo intradermally once weekly for 12 weeks, with safety, tolerability, and pharmacodynamic responses assessed over a 63-week period, including early-phase clinical effects assessed via HDM exposure in an allergen challenge chamber.

**Results:**

Twenty-eight adults (mean age, 26.9 years; 23 male participants), with 7 receiving 1 mg and 13 receiving 4 mg ASP2390, 8 receiving placebo, showed no serious adverse events or withdrawals due to treatment-emergent adverse events. The most common events were nasopharyngitis, coronavirus disease 2019, headache, fatigue, and diarrhea; fatigue and headache were the most frequent systemic reactions, and injection-site tenderness the most frequent local reaction. There were no substantial changes in allergen-specific immunoglobulin levels, basophil activation, or T helper cell subpopulations, and no difference in allergic clinical responses compared to placebo.

**Conclusion:**

Intradermal DNA vaccine ASP2390 is safe and well tolerated but does not show an immunologic or clinical response in a small sample of adults allergic to HDM.

The increasing prevalence of allergic respiratory diseases has become a major global public health concern.^1^ House dust mites (HDMs) are the primary perennial indoor allergen contributing to this condition and have been implicated in triggering allergen-associated asthma and rhinitis.^2^

Among the known *Dermatophagoides pteronyssinus* (Der p) allergen spectrum, Der p 1 and Der p 2 are the major allergens, triggering specific IgE responses in more than 80% of patients with HDM allergy. Moreover, Der p 7 and Der p 23 constitute significant additional allergens.^3^, ^4^, ^5^, ^6^

For airborne allergic diseases, current management focuses on allergen avoidance, symptom alleviation through pharmacotherapy, and allergen-specific immunotherapy (AIT). In the case of HDM allergy, cleaning measures like special mattress encasings, frequent washing of bed clothing, filtration, ventilation, and vacuuming are implemented, but their effectiveness is uncertain.^7^^,^^8^

Symptomatic medications such as antihistamines, corticosteroids, and β_2_ agonists are used for immediate relief, while AIT involves subcutaneous or sublingual administration of HDM extracts to target underlying immunologic mechanisms and produce sustained disease-modifying effects. However, these therapies may cause serious adverse reactions, such as anaphylaxis, and require frequent applications for at least 3 years for maximum efficacy.^9^

Molecular approaches with recombinant allergens, such as DNA-based vaccines, may have potential benefits compared to allergen extract-based AIT.^10^, ^11^, ^12^ One such approach involves incorporating human lysosomal-associated membrane protein (LAMP)-1 into a plasmid vector encoding for the major allergens of interest. This DNA plasmid is then taken up by antigen-presenting cells to produce an allergen–LAMP-1 fusion protein. Because of LAMP-1, the chimeric protein is directed to cellular lysosomes, where the allergen is processed and complexed to MHC class II antigens for presentation to CD4^+^ helper T cells.^13^^,^^14^ It is hypothesized that the LAMP-based DNA vaccination enhances immunogenicity and causes a shift of the T_H_2 response toward a nonallergenic T_H_1 response.^15^ Furthermore, because the allergen protein is confined to the endosomal–lysosomal trafficking pathway, the chance of free allergen release is thought to be minimized, which potentially reduces the risk of atopic reactions.^16^

LAMP-based DNA vaccines have been previously developed for Japanese red cedar (JRC) pollinosis (Cry j 2) and food allergy to peanuts (Ara h 1, 2, 3).^17^^,^^18^ Phase 1 trial results suggested the vaccine to be safe and potentially effective in treating JRC-induced allergy, with the majority of participants experiencing skin prick test (SPT) negative conversion after vaccination.^16^

ASP2390 is a novel LAMP-based DNA vaccine targeting the immune response to HDM allergens. It uses a single DNA plasmid encoding LAMP-1 as well as Der p 1, 2, 7, and 23 allergens and demonstrated nonclinical safety and efficacy in mice and rabbits. The objective of the present first-in-human study was to evaluate the safety, tolerability, and immunologic response to ASP2390 in adults allergic to HDMs. To assess clinical effects at an early phase, participants were exposed to HDM extracts in the Fraunhofer allergen challenge chamber.^19^

## Methods

This was a randomized, participant- and investigator-blinded, placebo-controlled, multiple ascending intradermal dosing, phase 1 study, which enrolled patients at 2 study sites in Germany (Fraunhofer ITEM, Hannover; and Parexel, Berlin) between September 2020 and December 2022. Allergen challenges were performed for all participants centrally at the Fraunhofer Institute in Hannover. The lead ethics committee of Hannover Medical School and the competent authority (Paul-Ehrlich-Institute) approved the research protocol. This study was registered before enrollment at ClinicalTrials.gov (NCT04184895).

### Study population

Adult male or female participants between 18 to 65 years of age with a history of HDM-induced allergic rhinitis (with or without conjunctivitis) were enrolled onto this study. For study inclusion, participants must have experienced a positive SPT response to Der p, a Der p–specific IgE level of ≥0.7 kU/L, and a total nasal symptom score (TNSS) of ≥6 at least twice during the last 3 hours within the 4-hour allergen chamber challenge test.

Participants with concomitant allergies to seasonal aeroallergens that were anticipated to become active within the study period and participants with concomitant allergies to animal dander (who had respective exposure on a regular basis) were excluded from the study. In addition, participants who received immunosuppressive treatment or immunotherapy treatment were also excluded from the study. Complete eligibility criteria can be found in Table E1 in this article’s Online Repository available at www.jaci-global.org.

### Study treatment

The study drug ASP2390, a 4 mg/0.4 mL solution for injection, was developed and supplied by Astellas. It consisted of a single plasmid multivalent Der p 1, 2, 7, and 23 vaccine along with phosphate-buffered saline in sterile, single-use vials. For placebo dosing, 0.9% sodium chloride solution was administered in a volume and route corresponding to the appropriate ASP2390 dose. Both ASP2390 and placebo were administered using the Terumo Immucise intradermal injection device. Because of the distinctive viscosity of ASP2390, designated unblinded study staff administered the study drug to maintain blinding.

An auxiliary medicinal product, Der p allergen (SQ 503, ALK, Hamburg, Germany), was dissolved in “10% m/V Lactose in isotonic saline solution” (Fraunhofer ITEM, Pharmaceutical Biotechnology, Braunschweig, Germany) and was nebulized by spray drying as “Der p allergen lactose solution” to induce symptoms of allergic rhinitis in participants in the allergen challenge chamber.^20^

### Study design

A series of 2 different dose groups was planned. The first cohort was to receive 1 mg of ASP2390 per dose and participant, while the second cohort was to receive 4 mg ASP2390. This dose could have been adjusted by the dose escalation committee on the basis of emergent safety and tolerability data, which were continuously assessed throughout the trial.

**Fig 1:**
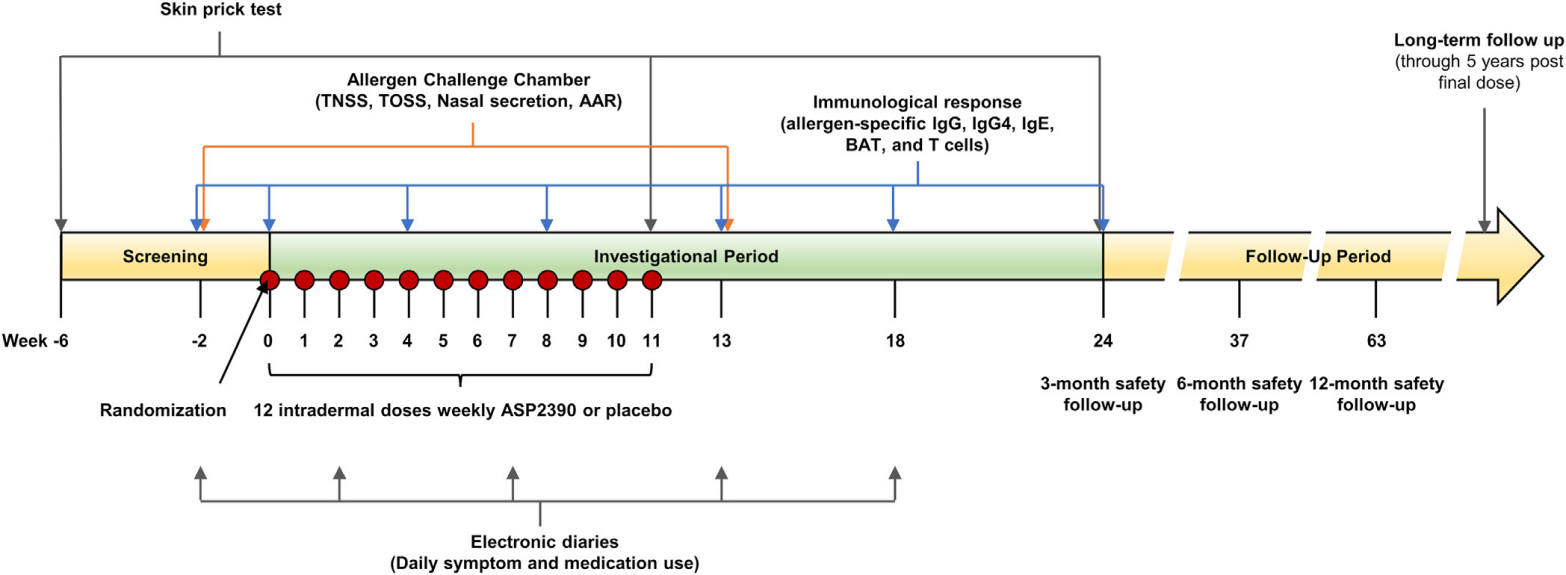
Study scheme. Study consisted of 3 periods: screening, investigation, and follow-up. After assessing eligibility and conducting a qualifying/baseline allergen chamber test, randomization to treatment with ASP2390 or placebo occurred at week 0. This was followed by weekly dose provided for 12 weeks. Primary study period concluded at week 63. Long-term safety follow-up will be conducted yearly for up to 5 years (until approximately December 2026). *AAR,* Active anterior rhinomanometry.

Participants were screened up to 6 weeks before enrollment (Fig 1). The initial screening visit assessed primary eligibility, including SPT and allergen-specific IgE. Once all eligibility criteria were confirmed, a qualifying allergen chamber test was conducted at a second screening that was used as pretreatment baseline. Randomization to treatment with ASP2390 or placebo occurred on day 1 within each cohort. After randomization, participants received intradermal doses of ASP2390 or placebo once every week for a total of 12 doses, using sentinel dosing for each cohort. Response assessments were conducted at the given time points (Fig 1). The primary study period concluded at week 63, approximately 1 year after the final study drug administration. After the final visit, all participants will be contacted yearly for long-term safety follow-up through 5 years after final dose, until approximately December 2026.

### Safety and tolerability assessments

The primary end point encompassed an array of safety and tolerability measures, which included the nature, frequency, and severity of treatment-emergent adverse events (TEAEs) reported in each dosing group. This evaluation also comprised safety measures such as hematologic, biochemical, and urinalysis parameters, as well as monitoring of vital signs, electrocardiograms, and physical examinations. To minimize risk during the chamber challenge, spirometry and peak expiratory flow measurements were assessed.

Systemic reaction events were evaluated directly for 1 hour after injection and graded using the World Allergy Organization Subcutaneous Immunotherapy Systemic Reaction Grading System.^21^ Next, both systemic and local reactogenicity events were evaluated from 1 hour after dosing up to 7 consecutive days after each injection, with participants recording their assessments in an electronic diary for each of the 7 days.^22^

Circulating anti–LAMP-1 antibody levels were monitored as an additional safety criterion.

### Pharmacodynamic assessments

Pharmacodynamic assessments were conducted as indicated in Fig 1. The secondary end point, Der p–specific IgG_4_, along with a panel of exploratory immunologic end points, was quantified using standardized assays. Specifically, Der p and *Dermatophagoides farinae* (Der f)-specific IgG_4_, IgG, and IgE levels were measured using the ImmunoCAP system, basophil activation was quantified with the Flow CAST basophil activation test (BAT), and allergen-reactive T-cell frequencies were identified by flow cytometry as described previously.^23^

SPT for Der p was performed at screening to assess eligibility and twice after vaccination to reveal a possible negative conversion (wheal diameter, <3 mm). For in-depth investigation of clinical response, participants were exposed to nebulized Der p allergen in a 4-hour allergen chamber challenge at screening and week 13. The experimental setting had been successfully validated and used in clinical studies before.^20^^,^^24^ Symptom scores in response to allergen exposure were monitored accordingly, including TNSS and total ocular symptom score (TOSS). Score kinetics stabilize after an initial phase of surge, so only the mean values of the last 2 hours of the challenge were used for outcome assessment. The weight of nasal secretions was assessed every 60 minutes during the 4-hour allergen challenge. At the same time points, active anterior rhinomanometry was measured, using the sum of flow rates at 150 Pa for each nostril as outcome measure.

Throughout the study, electronic diaries were used to evaluate symptom scores (daily rhinitis and conjunctivitis symptoms) and symptom-relieving medication receipt.^25^ Participants were asked to record the corresponding scores for 14 consecutive days starting the day after visits at screening 2, as well as weeks 2, 7, 13, and 18.

### Statistical analysis

No formal statistical sample size calculation has been performed for this phase 1 study. The sample size of 10 participants for cohort 1 (n = 7 ASP2390, n = 3 placebo) and 18 for cohort 2 (n = 13 ASP2390, n = 5 placebo) was considered sufficient to assess the objectives of the study. Early withdrawals could have been replaced. Descriptive statistics including frequency distributions and standard deviations (SDs) along with graphical representation were used to present and summarize the end points. Inferential statistical methods were not planned or conducted.

## Results

### Study population and exposure

A total of 234 participants provided informed consent, 206 of whom were removed from the study as a result of the stringent inclusion and exclusion criteria (see Table E1 in the Online Repository available at www.jaci-global.org), with inadequate reaction to HDM exposure in the screening allergen chamber and concomitant allergies to seasonal aeroallergens being the most frequent ones. Consequently, 28 participants were successfully randomized into the study. Specifically, 7 participants were assigned to the 1 mg ASP2390 treatment group, 13 participants were assigned to the 4 mg ASP2390 treatment group, and 8 participants were assigned to the placebo treatment group.

The study drug was administered intradermally once a week for a total of 12 administrations. Within the 4 mg ASP2390 treatment group, 2 participants received 11 instead of 12 doses. One missed dosing in week 3 and the other in week 4 because of a common cold and gastroenteritis, respectively. Apart from these instances, all other doses were administered as scheduled. All 28 participants successfully completed the study drug treatment, the investigational period, and the initial safety follow-up to week 63.

### Demographics and baseline characteristics

Participant age was between 18 and 36 years, with a mean age of 26.9 years. The total population comprised 23 male (82.1%) and 5 female (17.9%) participants. The body mass index ranged from 19.4 to 33.4 kg/m^2^, with a mean body mass index of 24.5 kg/m^2^. Most participants were White. Details are listed in Table E2 in the Online Repository available at www.jaci-global.org.

### Safety and tolerability results

During the primary study period, the 1 mg ASP2390 treatment group had 7 participants (100%) reporting a total of 55 TEAEs, the 4 mg ASP2390 treatment group had 13 participants (100%) reporting 87 TEAEs, and the placebo treatment group had 7 participants (87.5%) reporting 29 TEAEs. Among these TEAEs, a total of 10 in the 1 mg ASP2390 treatment group, 5 in the 4 mg ASP2390 treatment group, and 5 in the placebo treatment group were considered by the investigator to have a reasonable possibility of being caused by the study drug.

The most common TEAEs by preferred term (MedDRA v23.0), occurring in ≥4 participants in any treatment group, were nasopharyngitis, coronavirus disease 2019 (COVID-19), headache, fatigue, and diarrhea (Table I). A complete list of the incidence of all TEAEs is given in Table E3 in the Online Repository available at www.jaci-global.org. All reported TEAEs were considered mild or moderate in severity according to the investigator’s assessment. There were no deaths, serious adverse events, or TEAEs leading to withdrawal of treatment.

**Table I:** Summary of TEAEs

Characteristic	1 mg ASP2390	4 mg ASP2390	Placebo	Total
No. of participants	7	13	8	28
Overall TEAE				
Participants with any TEAE	7 (100.0)	13 (100.0)	7 (87.5)	27 (96.4)
Absolute no. of TEAEs	55	87	29	171
Participants with drug-related TEAEs∗	5 (71.4)	3 (23.1)	2 (25.0)	10 (35.7)
Absolute no. of drug-related TEAEs∗	10	5	5	20
Participants with serious TEAE	0	0	0	0
Participants with TEAE leading to therapy withdrawal	0	0	0	0
TEAEs in >15% participants by preferred term†				
Nasopharyngitis	2 (28.6)	8 (61.5)	1 (12.5)	11 (39.3)
COVID-19	0	8 (61.5)	3 (37.5)	11 (39.3)
Headache	4 (57.1)	4 (30.8)	3 (37.5)	11 (39.3)
Fatigue	1 (14.3)	2 (15.4)	3 (37.5)	6 (21.4)
Diarrhea	2 (28.6)	0	2 (25.0)	4 (14.3)
Upper respiratory tract infection	0	3 (23.1)	0	3 (10.7)
Back pain	2 (28.6)	1 (7.7)	0	3 (10.7)
Ear pain	0	2 (15.4)	0	2 (7.1)
Dysmenorrhea	0	2 (15.4)	0	2 (7.1)
Cough	0	2 (15.4)	0	2 (7.1)
Nasal congestion	2 (28.6)	0	0	2 (7.1)

Data are presented as nos. or nos. (%). All randomized participants received at least 1 dose of study drug.

∗ If investigator thought there was reasonable possibility of event being caused by study drug (or if relationship was missing), it was considered study drug related.

† Medical Dictionary for Regulatory Activities (MedDRA) v23.0. Participants could be counted in >1 category.

**Fig 2:**
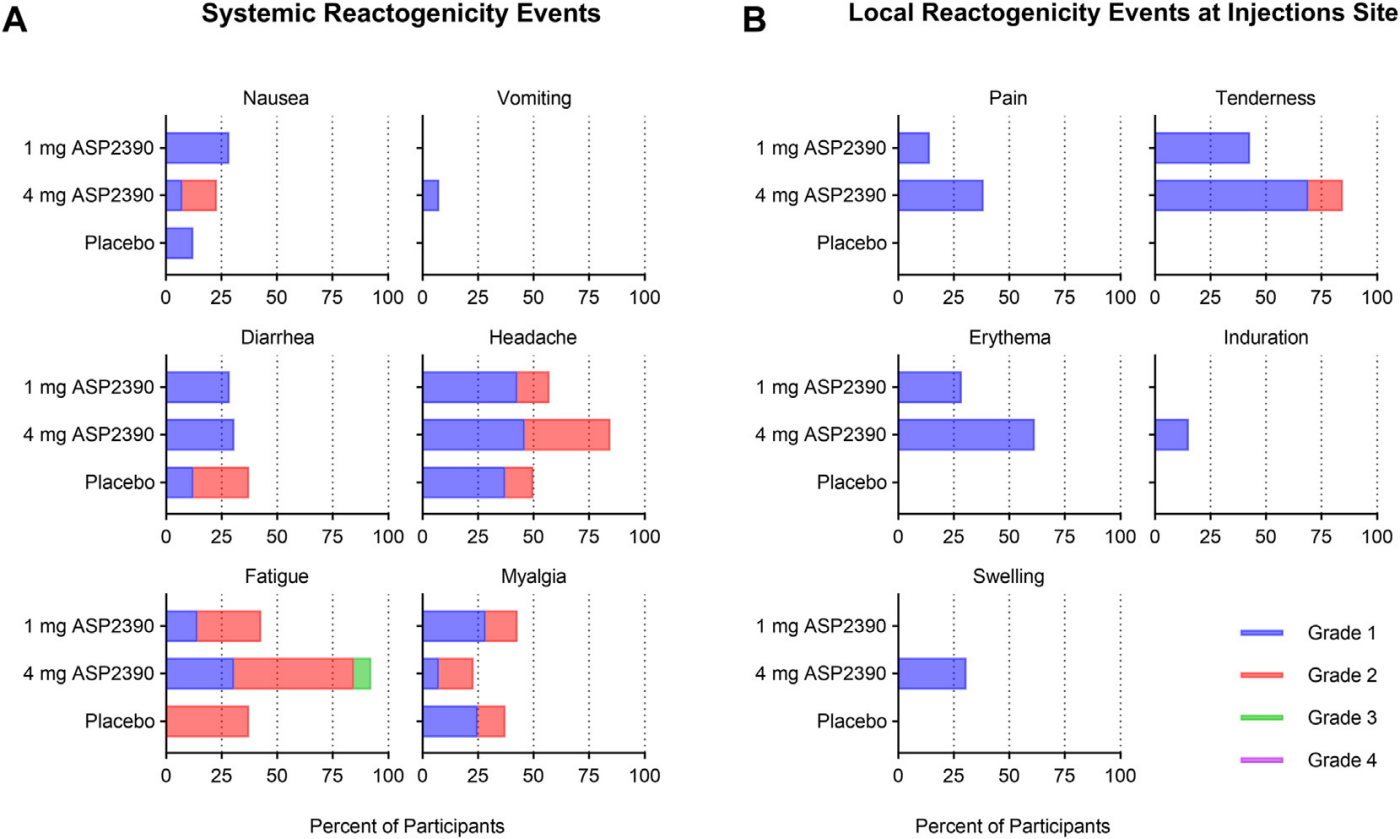
Systemic and local reactogenicity reactions. After each administration of study drug, reactions were monitored for 7 days. Here, participants were counted once, based on highest reported grade during each 7-day period. **(A)** Percentage of participants with systemic reactions (nausea, vomiting, diarrhea, headache, fatigue, myalgia). **(B)** Percentage of participants with local reactions (pain, tenderness, erythema, induration, swelling) at injection site. Each event was graded by Reactogenicity Grading Scale as follows: mild (grade 1), moderate (grade 2), severe (grade 3), and potentially life-threatening (grade 4).^22^

No participant experienced World Allergy Organization subcutaneous immunotherapy systemic reaction events within 60 minutes after the injection. Systemic and local reaction grades were reported for 7 days after each dosing. The most frequently reported systemic reactogenicity reaction (based on the cumulative number of events for all treatment groups) was fatigue, followed by headache. Only one participant in the 4 mg ASP2390 treatment group experienced a grade 3 systemic reaction of fatigue. This participant also had a TEAE of upper respiratory tract infection at the same time, which was determined by the investigator to be unrelated to the study drug. Among the local reactions reported (based on the cumulative number of events for all treatment groups), injection-site tenderness was the most common, followed by redness and pain. All local reactogenicity reactions were either grade 1 or grade 2 in severity (Fig 2).

Throughout the entire study duration, all participants tested negative for anti–LAMP-1 antibodies.

### Pharmacodynamic results

**Fig 3:**
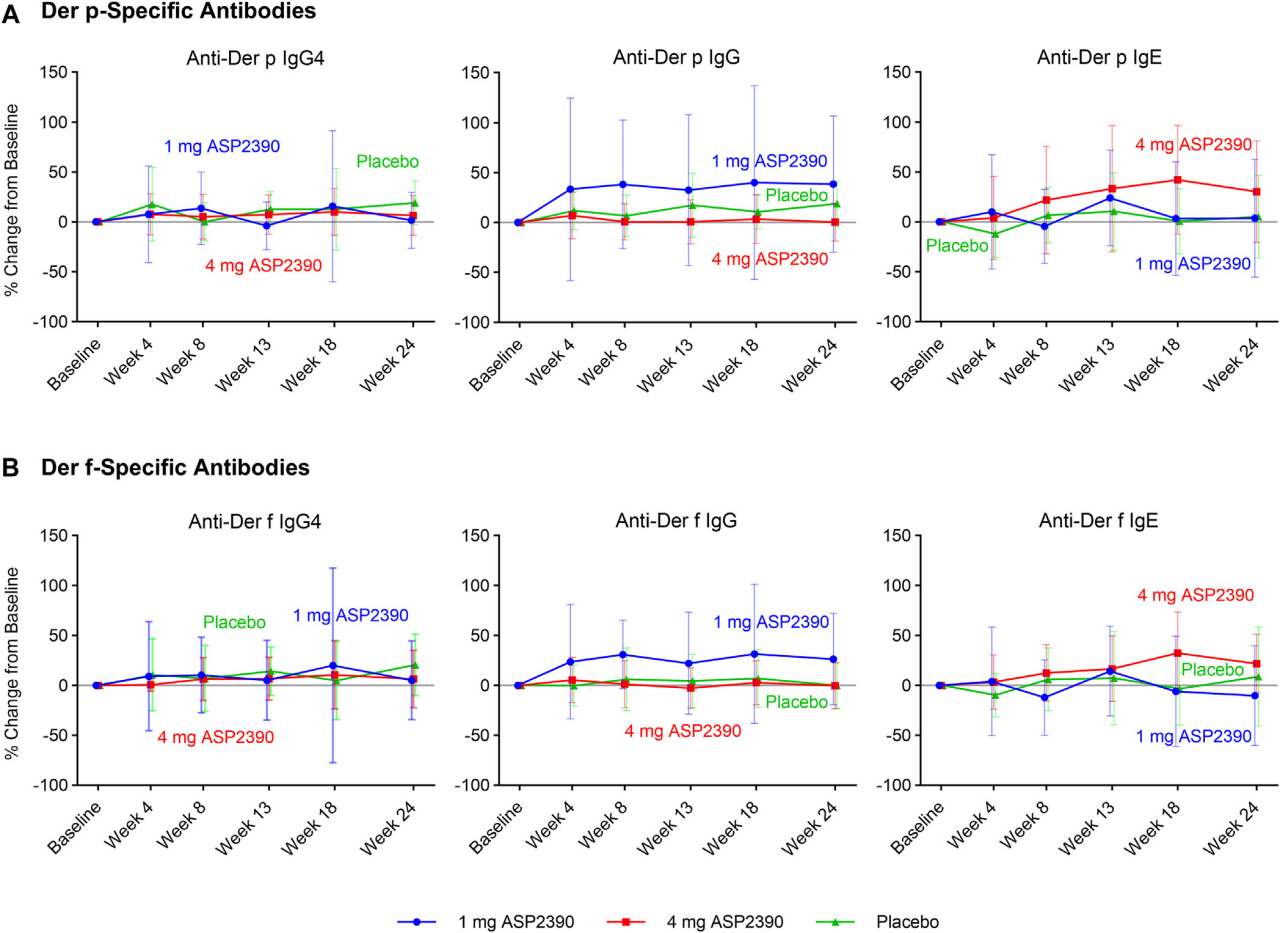
Immunologic response across different time points after study drug administration. Change from baseline (%) of IgG_4_*(left),* IgG *(center),* and IgE *(right)* antibodies against Der p **(A)** and Der f **(B)**. Data are shown as means ± SDs.

The mean results for allergen-specific IgG_4_, IgG, and IgE antibodies were similar across the 1 mg ASP2390, 4 mg ASP2390, and placebo treatment groups at different time points (Fig 3). Changes generally fell within the range observed in the placebo group, and there was no dose relationship observed. However, the 1 mg ASP2390 treatment group showed minor increases in mean percentage changes from baseline for allergen-specific IgG antibodies compared to the 4 mg ASP2390 and placebo treatment groups. There was no difference in specific antibody profiles for anti–Der p and anti–Der f.

In the BAT using both Der p and Der f, the mean results varied across all treatment groups and time points, with no specific trend observed in any group (for Der p, see Fig E2 in the Online Repository available at www.jaci-global.org).

Circulating HDM allergen–reactive T cells were detected in some samples, but not all. Generally, the HDM allergen–reactive T-cell subsets were similar to the placebo treatment group after dosing in all ASP2390 treatment groups (see Fig E3 in the Online Repository available at www.jaci-global.org).

**Fig 4:**
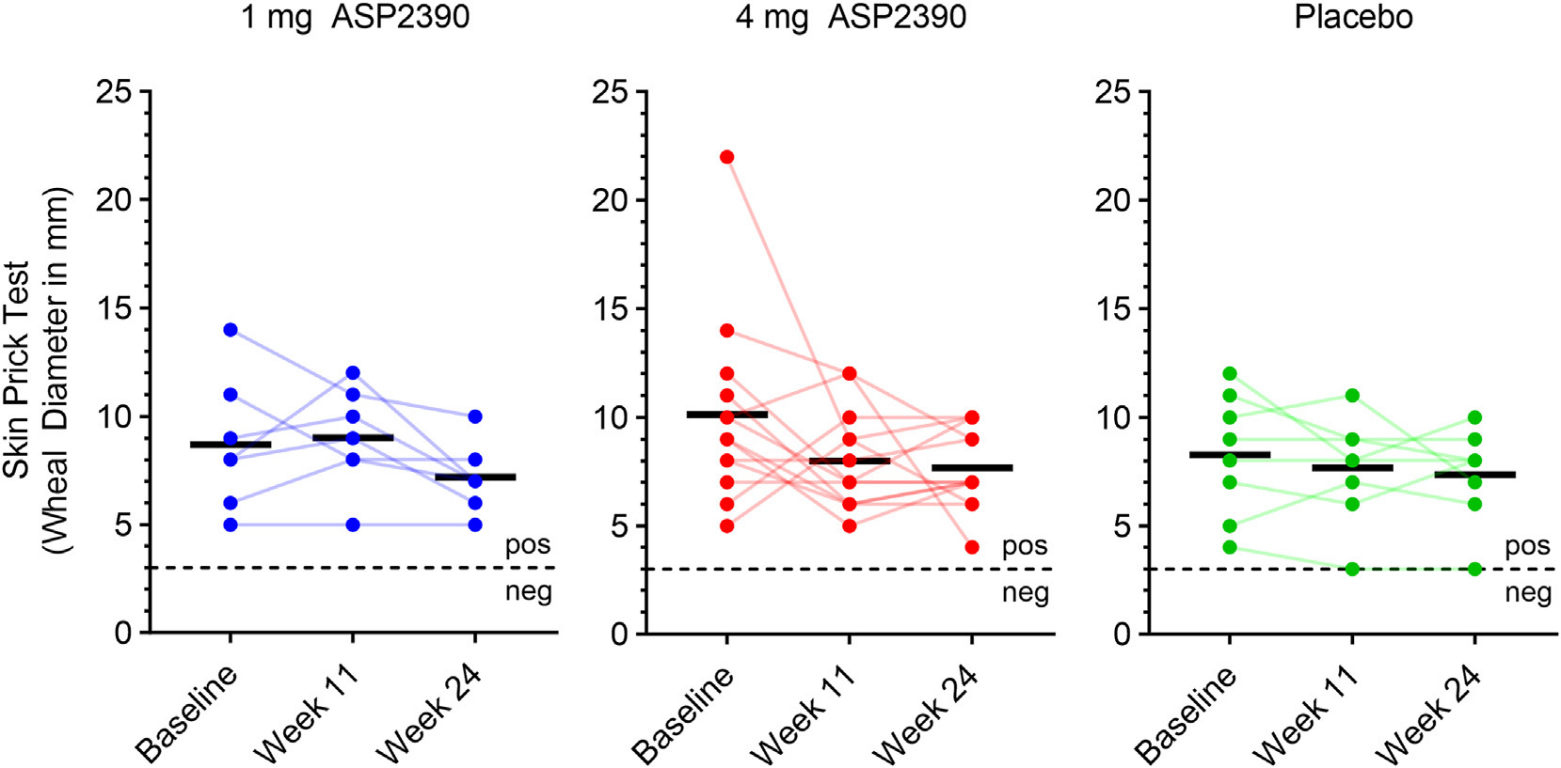
SPT results for Der p. Wheal diameter in mm is plotted against different time points for each participant. *Black bars* represent mean value; *dotted line,* threshold for possible negative conversion (<3 mm).

There was a slight decrease in SPT Der p wheal diameter in all groups, but this trend was not substantial, especially because none of the participants experienced a negative conversion (wheal diameter, <3 mm) after drug administration (Fig 4). The mean (SD) change from baseline for the SPT Der p wheal diameter in mm was 0.3 (2.6) at week 11 and −1.6 (1.7) at week 24 for the 1 mg treatment group, −2.1 (4.5) at week 11 and −2.5 (5.6) at week 24 for the 4 mg treatment group, and −0.6 (1.8) at week 11 and −0.9 (1.5) at week 24 for the placebo treatment group.

**Fig 5:**
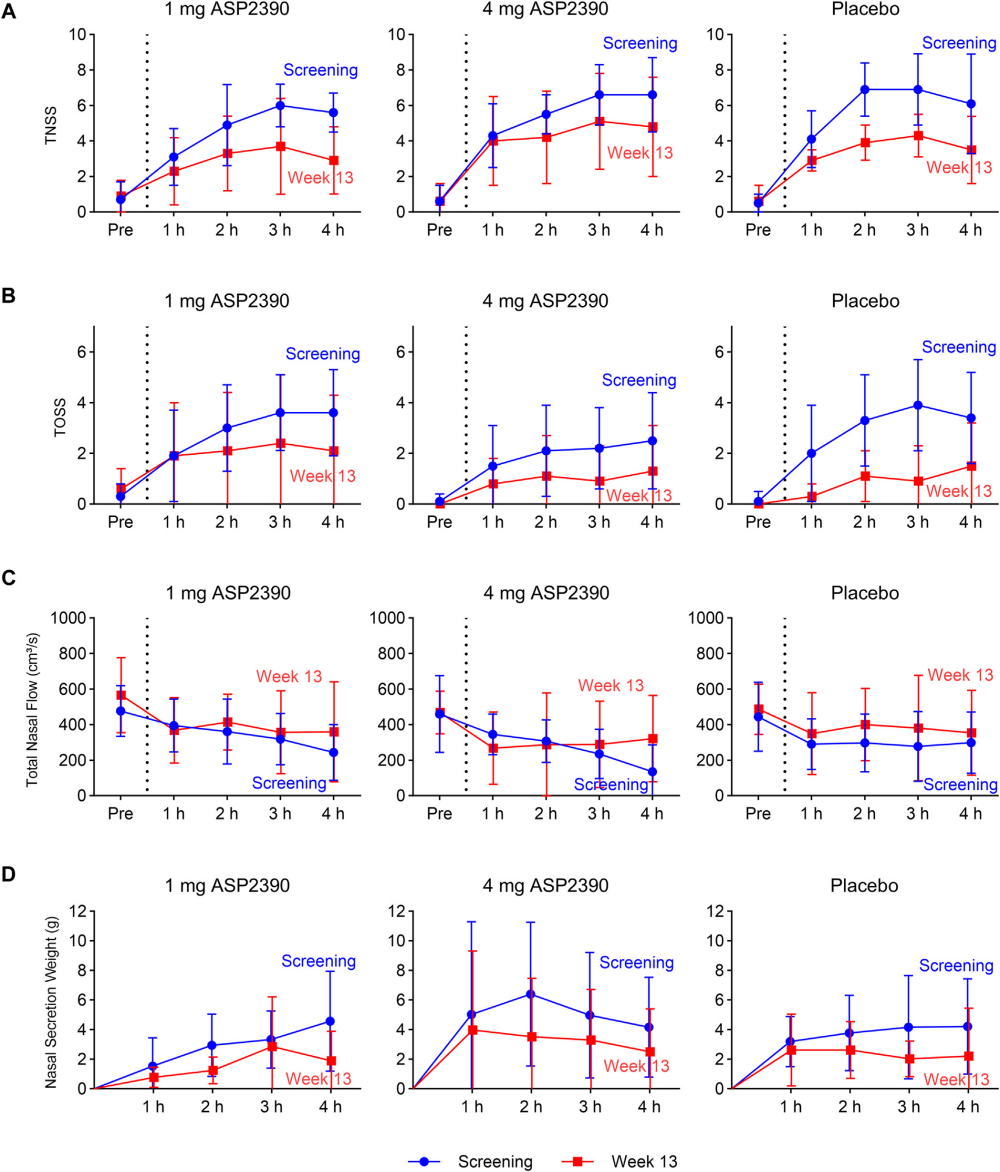
Responses in allergen challenge chamber. **(A)** TNSS, **(B)** TOSS, **(C)** total nasal flow (cm³/s), and **(D)** nasal secretion weight (g). Data are presented as means (±1 SD) before allergen challenge (Pre) and hourly during allergen challenge, comparing baseline (screening, *blue lines*) with week 13 *(red lines). Dotted lines* in *(A-C)* indicate initiation of Der p exposure.

As an exploratory efficacy readout, participants were exposed to nebulized Der p in the allergen challenge chamber, and the mean changes in TNSS and TOSS from screening baseline were assessed at week 13. For TNSS, the mean change from baseline was −2.14 (1.61) for the 1 mg ASP2390 group, −1.40 (1.78) for the 4 mg ASP2390 group, and −3.02 (1.39) for the placebo group. For TOSS, the mean change from baseline was −1.29 (1.15) for the 1 mg ASP2390 group, −1.08 (1.91) for the 4 mg ASP2390 group, and −2.54 (1.49) for the placebo group. Overall, all treatment groups showed a decrease in nasal and ocular symptom scores from baseline to week 13. However, there was no meaningful difference observed between treatments with ASP2390 or placebo (Fig 5, *A* and *B*). In line with improvement of subjective symptoms, there was an increase in nasal flow objectively measured by rhinomanometry between baseline and week 13 across all treatment groups. Complementarily, nasal secretion was reduced in all groups (Fig 5, *C* and *D*).

Electronic diaries were utilized to assess daily symptom scores and medication receipt. Throughout the study period, a slight decrease in daily symptom scores was observed. However, no substantial difference was observed between treatment with ASP2390 or placebo (see Fig E4, *A,* in the Online Repository available at www.jaci-global.org). The daily medication score remained relatively stable, with little change from baseline throughout the study period and no difference between treatment groups (Fig E4, *B*).

## Discussion

### Safety and tolerability

This first-in-human study provides evidence for safety and tolerability of intradermal HDM DNA vaccine ASP2390. All reported TEAEs were mild to moderate. No deaths, serious adverse events, or withdrawals due to TEAEs occurred. Also, no anti–LAMP-1 antibodies were observed. As described, a major advantage of the LAMP-Vax platform is that because the allergen processing is confined to the endosomal–lysosomal trafficking pathway, no free allergen is released. Our safety data appear to provide additional evidence for this concept, as there were no indications of atopic or anaphylactic reactions.

Systemic reactogenicity was mainly characterized by fatigue and headache, with no significant difference between placebo and the 1 mg dose of ASP2390. However, the 4 mg dose group experienced both symptoms more frequently, including one case of grade 3 fatigue, which was potentially confounded by an upper respiratory tract infection. In this context, we note that the study was conducted amid the COVID-19 pandemic. Of the 28 participants enrolled, a total of 11 were infected with severe acute respiratory syndrome coronavirus 2 during the course of the study. Of note, we had no evidence that such an infection substantially influenced the immune response to ASP2390.

Local reactions, such as injection-site tenderness, erythema, and pain, were more prevalent in the 4 mg dose group compared to the 1 mg group, indicating a possible dose-dependent response. No local reactions were reported in the placebo group.

### Dose selection and interval justification

Preclinical studies and a quantitative systems pharmacology model guided both dose and interval selection, predicting increased allergen-specific IgG production with 1 to 4 mg of ASP2390. As a result of injection constraints, 4 mg weekly was the highest feasible dose. The quantitative systems pharmacology model suggested weekly dosing would be most effective for this phase 1 study. The 12-week duration balanced biomarker assessment and clinical efficacy within a manageable time frame. Although longer dosing intervals might yield different outcomes, this study aimed to demonstrate potential effects for the most frequent and highest feasible dose before considering extended regimens in future phases.

### Missing pharmacodynamic response

As encouraging as the safety data were, unfortunately, this was not translated into a corresponding effectiveness. A broad set of functional immunologic tests including SPT, allergen-specific immunoglobulin levels (IgG_4_, IgG, and IgE), BAT, and T-cell responses were analyzed but failed to show clear evidence for pharmacodynamic response.

Although *in vitro* studies using HEK293T cells confirmed antigen expression, *in vivo* verification for ASP2390 presented technical challenges. Therefore, allergen-specific antibodies were used as a surrogate. In mice treated with 50 μg provided subcutaneously weekly, levels of Der p 1, 2, 7, and 23–specific IgG_1_ and IgG_2_a increased compared to control. However, the lack of efficacy in this clinical trial may also be due to the use of naked plasmid DNA, as opposed to the *in vitro* studies that used lipofectamine to enhance plasmid uptake. This may have resulted in low plasmid uptake by antigen-presenting cells *in vivo,* contributing to the observed low immune response.

In terms of clinical response, Su et al^16^ demonstrated a notable SPT negative conversion in the majority of participants receiving the CryJ2-LAMP plasmid vaccine against JRC aeroallergen, whereas our study observed only a slight numerical decrease in SPT response to HDM without any complete negative conversions. One could argue that the SPT negative conversions observed by Su et al were partially influenced by the absence of natural JRC exposure in Hawaii for the Japanese expatriates included in the trial. However, because of the lack of a placebo group in the respective study, a definitive answer to this question cannot be provided.

### Pronounced placebo effect

The clinical response showcased a pronounced placebo effect, which seemed to outweigh any effect size of the investigational drug. Both TNSS and TOSS substantially decreased in response to HDM exposure in the allergen chamber after vaccination compared to pretreatment baseline, predominantly in the placebo group. Similarly, daily symptom scores decreased across all groups.

AIT trials are often flawed by substantial placebo effects, which can diminish or even negate the presumed efficacy of the investigational drug.^26^^,^^27^ The European Academy of Allergy and Clinical Immunology task force paper on the placebo effect in AIT trials recommends the implementation of rhinomanometry as a non- or less placebo–susceptible outcome.^28^ However, in the present study, a placebo effect was also detected for nasal flow and nasal secretion weight as objective outcome measures. In general, repeatability of objective and subjective outcome measures in the challenge chamber was found to be excellent,^29^ thus justifying study designs with chamber repetitions, and therefore clearly identifying that placebo treatment also affects objective outcome measures such as nasal flow and nasal secretion weight in AIT trials. Of note, placebo effects on objective outcome measures have also been described in the chamber setting for non-AIT trails,^30^ although to a much smaller extent. The present study was not set up to perform inferential statistics on these exploratory findings, so the significance of these results is uncertain but merits further investigation.

### Limitations

Despite a complex and ambitious trial design aiming at detecting efficacy signals at an early development stage, this study carries several limitations. First, the number of study participants was small, and the sample size was not powered for hypothesis testing. However, participant numbers are in line with standard phase 1 designs and sufficient to detect relevant safety signals. Despite the small sample size, we added multiple immunologic and experimental clinical outcome measures to the study procedures to deepen our understanding of the treatment’s implications. Second, ASP2390 might have failed to demonstrate relevant efficacy as a result of its limited coverage of HDM major allergens. However, the most relevant Der p allergens for the HDM patients in Europe^4^ were represented, and patient selection was done accordingly. Last, as a result of technical challenges associated with detecting protein antigen *in vivo,* it was not possible to test the ASP2390 vaccine before study conduct to prove the level of allergen peptide–major MHC expression on human antigen-presenting cells and its duration of expression.

### Challenges, setbacks, and perspectives

The use of the LAMP platform in the creation of novel AIT treatments initially appeared to be a breakthrough, offering a solution to the drawbacks of lengthy treatments with crude allergen extracts. Yet the reality has proven to be even more challenging, as evidenced by the now-raised efficacy data for ASP2390. These results are in line with other setbacks within the LAMP platform for AIT, including the discontinuation of ASP4070 in phase 2 for lack of efficacy in treating JRC pollinosis (NCT03101267) and the termination of ASP0892 in phase 1 for peanut allergy (NCT02851277, NCT03755713), which, despite a favorable safety profile, did not prove efficacious.^23^

Our first-in-human study with ASP2390 is a reminder of the nuanced and unpredictable nature of AIT drug development. Although its application is safe, the study lacked evidence for efficacy. Thus, even with promising new approaches, there remains an urgent need for more research and innovative studies aimed at discovering and refining alternative therapies that can provide tangible benefits to suffering allergy patients.

**Key messages**

• ASP2390 is a novel LAMP-based DNA vaccine targeting key HDM allergens.
• Our first-in-human study confirms the safety and tolerability of intradermal ASP2390.
• No immunologic or clinical response was observed in a small sample of adults with HDM-induced allergic rhinitis, so the study lacked evidence for efficacy.

## Disclosure statement

Sponsored by 10.13039/100007705Astellas Pharma Global Development Inc.

Disclosure of potential conflict of interest: J. M. Hohlfeld declares grants to his institution from Astellas for this clinical trial; grants to his institution for clinical trial conduct from Altamira, AstraZeneca, Bayer AG, 10.13039/100008349Boehringer Ingelheim Pharma, CSL Behring, Desitin Arzneimittel, EpiEndo, F. Hoffmann-La Roche, Genentech, OM Pharma, Novartis, ReAlta Life Sciences, Sanofi-Aventis Deutschland; and personal fees for consultancy and board activities from Boehringer Ingelheim Pharma, Celerion, CSL Behring, Cureteq, Novartis, and Roche. E. Wambre and H. A. DeBerg declare research sponsorship to their institution from Astellas to perform allergen-specific T-cell assays for this study. R. Smulders, T. Kusawake, G. R. Chichili, M. B. Blauwet, A. Spence, M. Patton, R. Tabash, and B. C. Ferslew are employees of Astellas Pharma Global Development Inc. The rest of the authors declare that they have no relevant conflicts of interest.

## References

[1] Pawankar R. (2014). Allergic diseases and asthma: a global public health concern and a call to action. World Allergy Organ J.

[2] Calderón M.A., Linneberg A., Kleine-Tebbe J., Blay F de, Hernandez Fernandez de Rojas Dolores, Virchow J.C. (2015). Respiratory allergy caused by house dust mites: what do we really know?. J Allergy Clin Immunol.

[3] Thomas W.R. (2015). Hierarchy and molecular properties of house dust mite allergens. Allergol Int.

[4] Weghofer M., Thomas W.R., Kronqvist M., Mari A., Purohit A., Pauli G. (2008). Variability of IgE reactivity profiles among European mite allergic patients. Eur J Clin Invest.

[5] Taketomi E.A., Silva D.A.O., Sopelete M.C., Gervásio A.M., Alves R., Sung S.J. (2006). Differential IgE reactivity to Der p 1 and Der p 2 allergens of *Dermatophagoides pteronyssinus* in mite-sensitized patients. J Investig Allergol Clin Immunol.

[6] Walsemann T., Böttger M., Traidl S., Schwager C., Gülsen A., Freimooser S. (2023). Specific IgE against the house dust mite allergens Der p 5, 20 and 21 influences the phenotype and severity of atopic diseases. Allergy.

[7] Wise S.K., Damask C., Roland L.T., Ebert C., Levy J.M., Lin S. (2023). International consensus statement on allergy and rhinology: allergic rhinitis—2023. Int Forum Allergy Rhinol.

[8] Gøtzsche P.C., Johansen H.K. (2008). House dust mite control measures for asthma: systematic review. Allergy.

[9] Pfaar O., Ankermann T., Augustin M., Bubel P., Böing S., Brehler R. (2022). Guideline on allergen immunotherapy in IgE-mediated allergic diseases: S2K guideline of the German Society of Allergology and Clinical Immunology (DGAKI), Society of Pediatric Allergology and Environmental Medicine (GPA), Medical Association of German Allergologists (AeDA), Austrian Society of Allergology and Immunology (ÖGAI), Swiss Society for Allergology and Immunology (SSAI), German Dermatological Society (DDG), German Society of Oto-Rhino-Laryngology, Head and Neck Surgery (DGHNO-KHC), German Society of Pediatrics and Adolescent Medicine (DGKJ), Society of Pediatric Pulmonology (GPP), German Respiratory Society (DGP), German Professional Association of Otolaryngologists (BVHNO), German Association of Paediatric and Adolescent Care Specialists (BVKJ), Federal Association of Pneumologists, Sleep and Respiratory Physicians (BdP), Professional Association of German Dermatologists (BVDD). Allergol Select.

[10] Zhernov Y., Curin M., Khaitov M., Karaulov A., Valenta R. (2019). Recombinant allergens for immunotherapy: state of the art. Curr Opin Allergy Clin Immunol.

[11] Su Y., Romeu-Bonilla E., Heiland T. (2017). Next generation immunotherapy for tree pollen allergies. Hum Vaccin Immunother.

[12] Durham S.R., Shamji M.H. (2023). Allergen immunotherapy: past, present and future. Nat Rev Immunol.

[13] Rowell J.F., Ruff A.L., Guarnieri F.G., Staveley-O’Carroll K., Lin X., Tang J. (1995). Lysosome-associated membrane protein-1–mediated targeting of the HIV-1 envelope protein to an endosomal/lysosomal compartment enhances its presentation to MHC class II–restricted T cells. J Immunol.

[14] Wu T.C., Guarnieri F.G., Staveley-O’Carroll K.F., Viscidi R.P., Levitsky H.I., Hedrick L. (1995). Engineering an intracellular pathway for major histocompatibility complex class II presentation of antigens. Proc Natl Acad Sci U S A.

[15] Su Y., Connolly M., Marketon A., Heiland T. (2016). CryJ-LAMP DNA vaccines for Japanese red cedar allergy induce robust Th1-type immune responses in murine model. J Immunol Res.

[16] Su Y., Romeu-Bonilla E., Anagnostou A., Fitz-Patrick D., Hearl W., Heiland T. (2017). Safety and long-term immunological effects of CryJ2-LAMP plasmid vaccine in Japanese red cedar atopic subjects: a phase I study. Hum Vaccin Immunother.

[17] Li X.M., Song Y., Su Y., Heiland T., Sampson H.A. (2015). Immunization with ARA h 1, 2, 3–Lamp-Vax peanut vaccine blocked IgE mediated anaphylaxis in a peanut allergic murine model. J Allergy Clin Immunol.

[18] Su Y., Heiland T., Connolly M., Marketon A. (2015). Lamp-based DNA vaccine for Japanese red cedar allergy. J Allergy Clin Immunol.

[19] Krug N., Badorrek P., Hohlfeld J.M. (2012). Experience with an allergen challenge chamber for clinical trials in allergic rhinitis. Clin Exp Allergy Rev.

[20] Lueer K., Biller H., Casper A., Windt H., Mueller M., Badorrek P. (2016). Safety, efficacy and repeatability of a novel house dust mite allergen challenge technique in the Fraunhofer allergen challenge chamber. Allergy.

[21] Cox L., Larenas-Linnemann D., Lockey R.F., Passalacqua G. (2010). Speaking the same language: the World Allergy Organization Subcutaneous Immunotherapy Systemic Reaction Grading System. J Allergy Clin Immunol.

[22] US Department of Health and Human Services; Food and Drug Administration; Center for Biologics Evaluation and Research (September 2007). Guidance for industry: toxicity grading scale for healthy adult and adolescent volunteers enrolled in preventive vaccine clinical trials. https://www.fda.gov/media/73679/download.

[23] Ferslew B.C., Smulders R., Zhu T., Blauwet M.B., Kusawake T., Spence A. (2024). Safety and immunopharmacology of ASP0892 in adults or adolescents with peanut allergy: two randomized trials. Allergy.

[24] Struß N., Dieter S., Schwarz K., Badorrek P., Hohlfeld J.M. (2023). Sodium chloride versus lactose as a carrier for house dust mite allergen in allergen chamber studies: a clinical study to assess noninferiority. Int Arch Allergy Immunol.

[25] Demoly P., Emminger W., Rehm D., Backer V., Tommerup L., Kleine-Tebbe J. (2016). Effective treatment of house dust mite–induced allergic rhinitis with 2 doses of the SQ HDM SLIT-tablet: results from a randomized, double-blind, placebo-controlled phase III trial. J Allergy Clin Immunol.

[26] van Gerth Wijk R. (2018). Positive and negative AIT trials: what makes the difference?. Allergo J Int.

[27] Wedi B., Wieczorek D., Kapp A. (2017). Placeboeffekt in Studien zur allergenspezifischen Immuntherapie mit Inhalationsallergenen. Hautarzt.

[28] Pfaar O., Agache I., Bergmann K.C., Bindslev-Jensen C., Bousquet J., Creticos P.S. (2021). Placebo effects in allergen immunotherapy—an EAACI Task Force position paper. Allergy.

[29] Hohlfeld J.M., Holland-Letz T., Larbig M., Lavae-Mokhtari M., Wierenga E., Kapsenberg M. (2010). Diagnostic value of outcome measures following allergen exposure in an environmental challenge chamber compared with natural conditions. Clin Exp Allergy.

[30] Badorrek P., Dick M., Schauerte A., Hecker H., Murdoch R., Luettig B. (2009). A combination of cetirizine and pseudoephedrine has therapeutic benefits when compared to single drug treatment in allergic rhinitis. Int J Clin Pharmacol Ther.

