# Supplementary material for: Phase 1 study of safety, tolerability, and efficacy of intradermal DNA vaccine ASP2390 in adults allergic to house dust mites
Source: J Allergy Clin Immunol Glob. 2025 Jan 7;4(2):100404. doi: 10.1016/j.jacig.2025.100404 (PMC11851213; doi:10.1016/j.jacig.2025.100404)
Supplement: Supplementary data [file mmc1.docx]

Supplementary Figure E1: Study Flow Diagram.

Enrollment and outcomes through week 63. For the 4 mg ASP2390 group, 2 participants received 11 instead of 12 doses: one missed dosing in week 3, the other in week 4. The rest of the dosages were administered as scheduled.

**
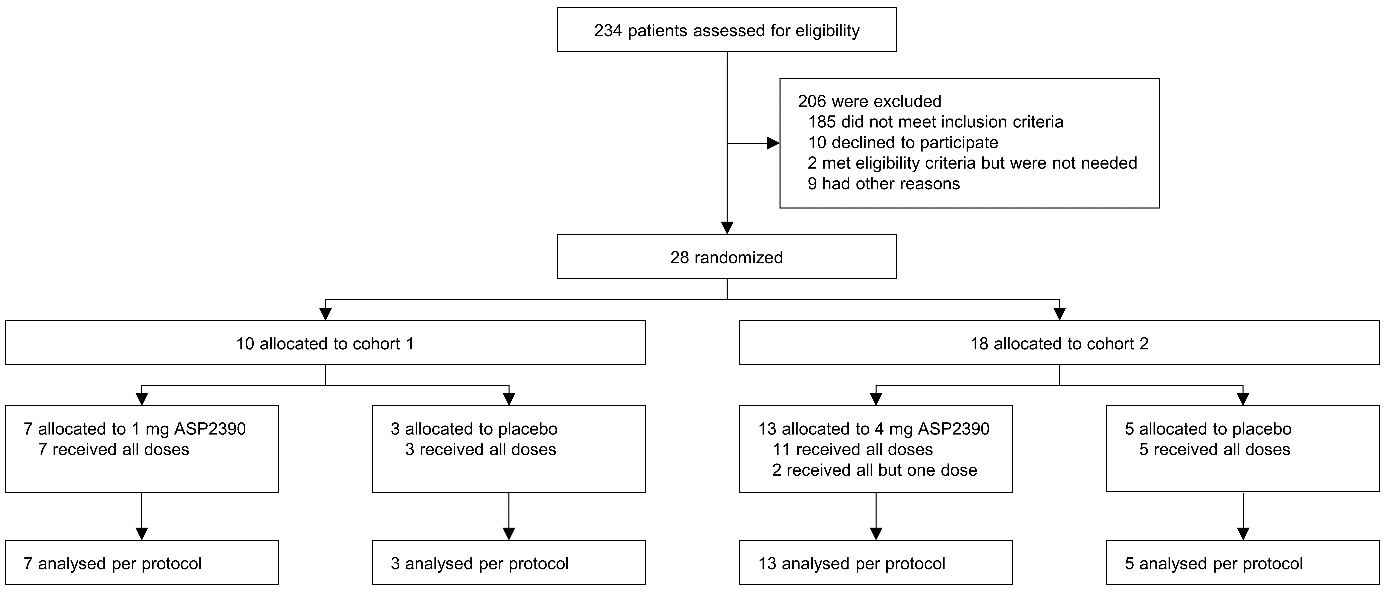
**

Supplementary Figure E2: Basophil Activation Test.

Percentage of CD63-positive basophils in response to Der p (D1) allergen at different dilutions (0.01 – upper panel; 0.1 – middle panel; 1 – lower panel) over the study period. Boxes show the 25th, 50th (median), 75th quartiles and mean (+ symbol). Whiskers are the max/min values within the data range or 1.5 times the inter-quartile range (IQR), whichever is smaller. Any values shown outside the whiskers are outliers that exceed 1.5 x IQR.

**
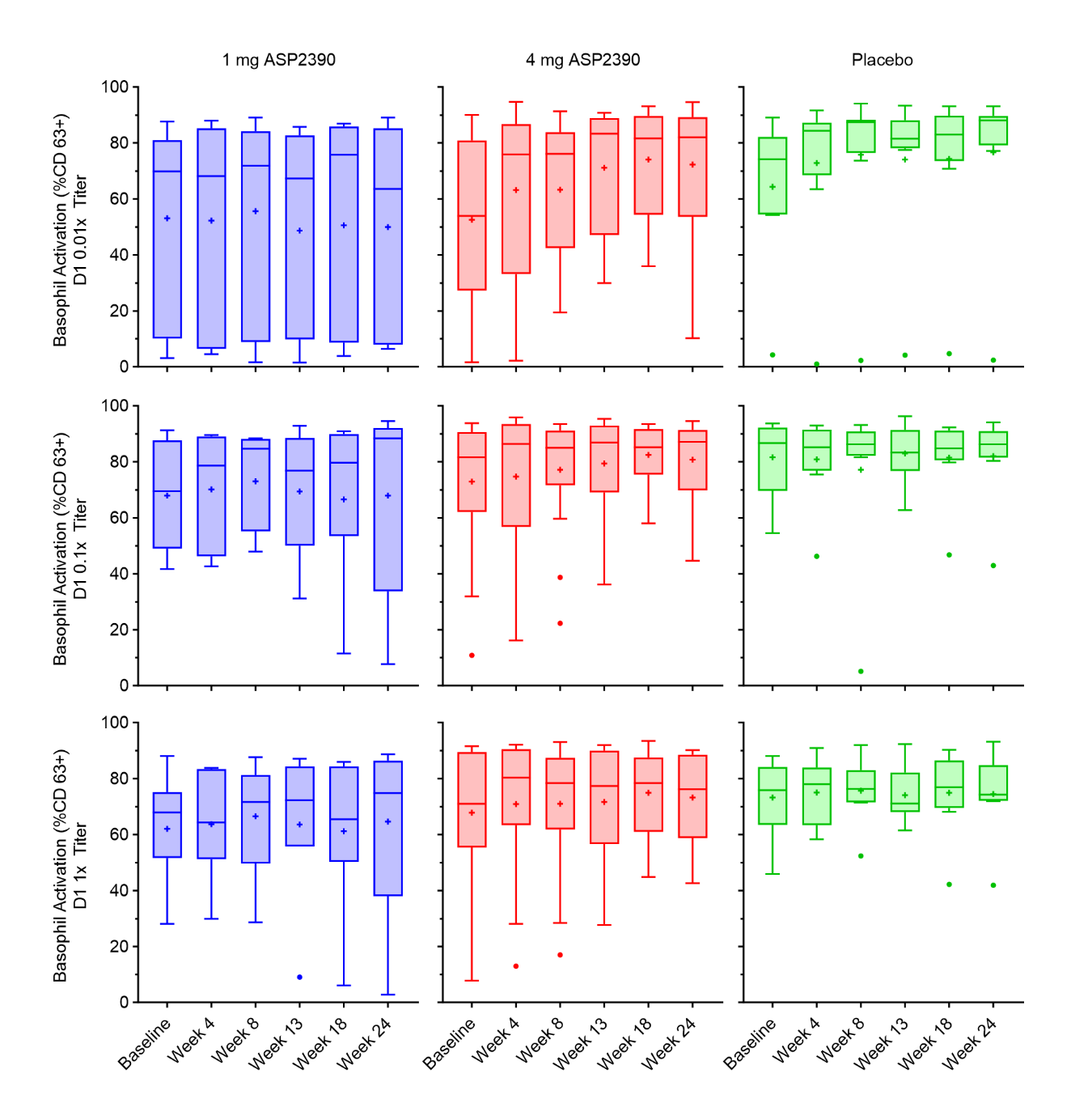
**

Supplementary Figure E3: HDM-specific T cells.

Proportion of HDM-specific TH2conv (Panel A), and TH2A cells (Panel B). All measurements taken are shown as black dots. In the majority of samples, fewer than 20 Der p reactive T-cells were detected and as a result, phenotyping could not be performed. Boxes show the 25th, 50th (median), 75th quartiles and mean (+ symbol). Whiskers are the max/min values within the data range or 1.5 times the inter-quartile range (IQR), whichever is smaller. Any values shown outside the whiskers are outliers that exceed 1.5 x IQR.

**
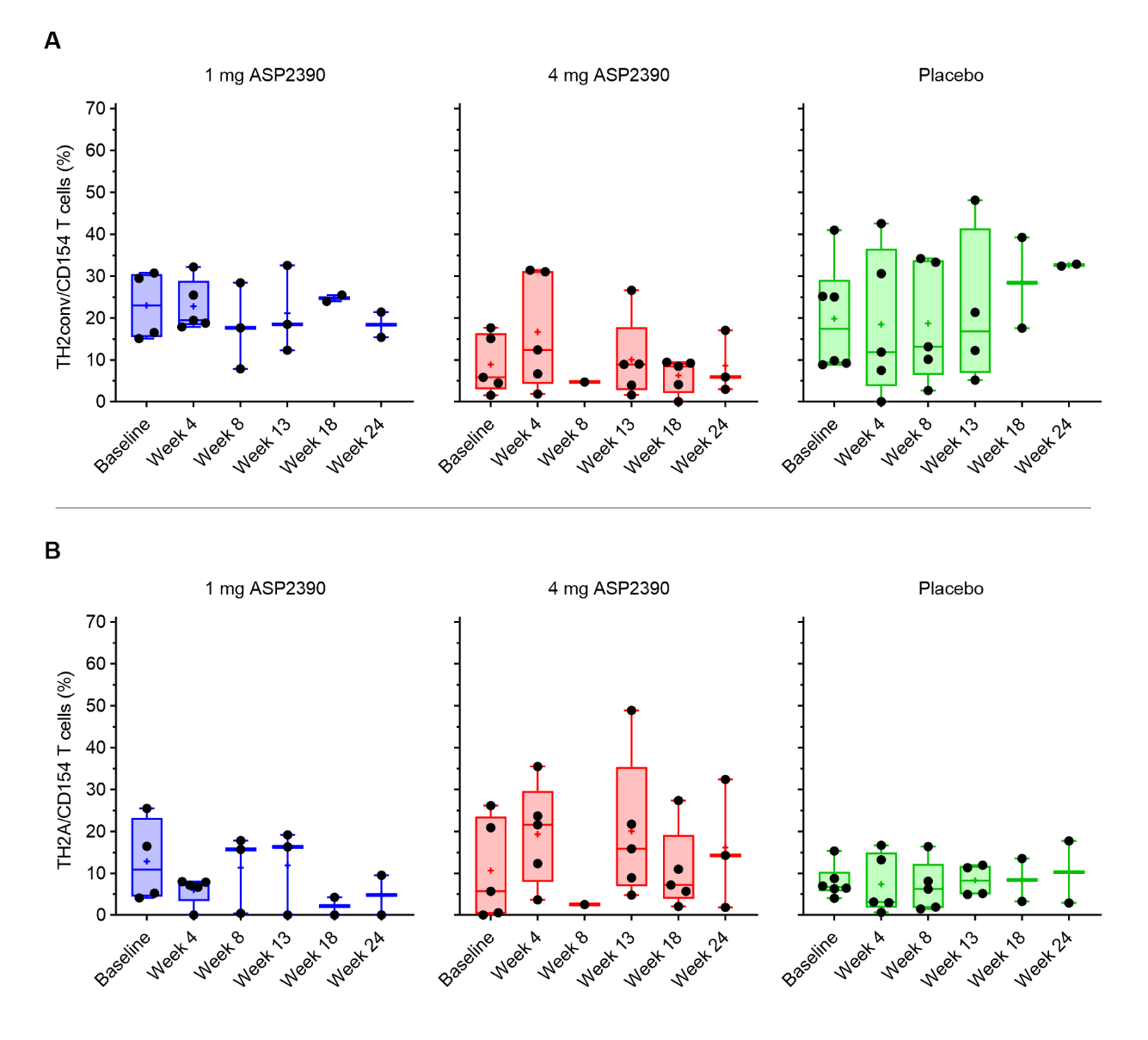
**

Supplementary Figure E4: Daily Symptom and Medication Score.

Participants were asked to record the daily symptom score and the daily medication score (according to Demoly et al. (1)) each day for 14 consecutive days, starting the day after the visits at screening 2, weeks 2, 7, 13 and 18. Mean change from baseline (± 1 SD) of A) Daily Symptom Score (DSS) and B) Daily Medication Score (DMS) per treatment group for allergic rhinitis domain (upper panels) and conjunctivitis domain (lower panels).


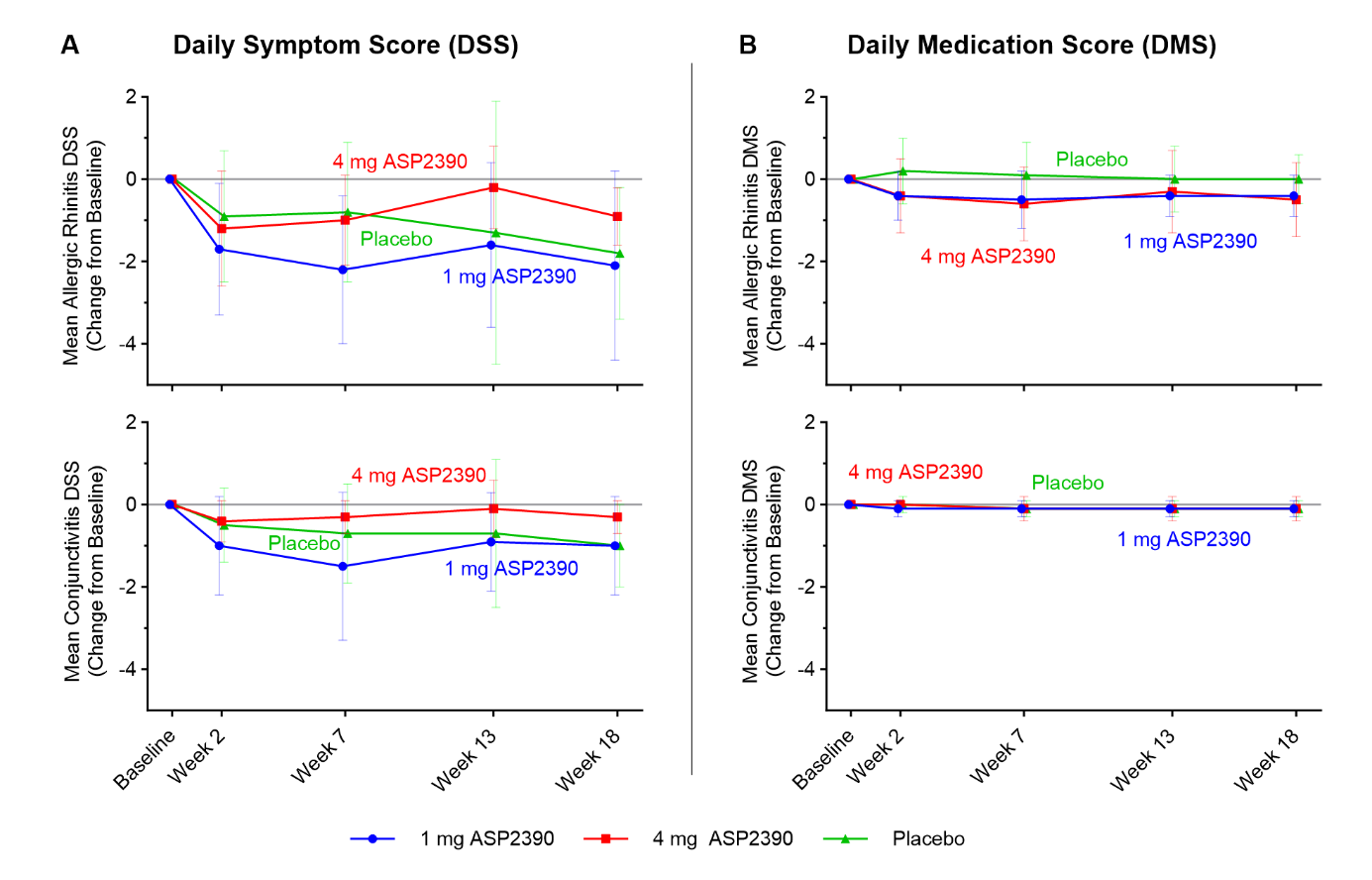


References

1. Demoly P, Emminger W, Rehm D, Backer V, Tommerup L, Kleine-Tebbe J. Effective treatment of house dust mite-induced allergic rhinitis with 2 doses of the SQ HDM SLIT-tablet: Results from a randomized, double-blind, placebo-controlled phase III trial. J Allergy Clin Immunol 2016; 137(2):444-451.e8.
