# Supplementary material for: Phase 1 study of safety, tolerability, and efficacy of intradermal DNA vaccine ASP2390 in adults allergic to house dust mites
Source: J Allergy Clin Immunol Glob. 2025 Jan 7;4(2):100404. doi: 10.1016/j.jacig.2025.100404 (PMC11851213; doi:10.1016/j.jacig.2025.100404)
Supplement: Supplementary data 1 [file mmc2.docx]

Supplementary Table E1: Criteria for inclusion/exclusion.

The study population consisted of adult male and female participants between 18 and 65 years of age with allergic rhinitis due to house dust mite (HDM). Eligible participants had to be able to give written informed consent and fulfil all inclusion and no exclusion criteria.

| **Inclusion Criteria** | Subject is eligible for the study if all of the following apply:   1. Institutional Review Board (IRB)/Independent Ethics Committee (IEC)-approved written informed consent and privacy language as per national regulations (e.g., Health Insurance Portability and Accountability Act Authorization for US sites) must be obtained from the subject prior to any study-related procedures (including withdrawal of prohibited medication, if applicable). 2. Subject is a male or female subject between 18 to 65 years of age, inclusive, at screening 1. 3. Subject has a history of HDM-induced allergic rhinitis with or without conjunctivitis of 1 year or longer in duration at screening 1. 4. Subject has positive SPT responses (positive wheal diameter reaction of ≥ 3 mm larger than the negative control and wheal diameter < 2 mm to the saline/diluent negative control) to D. pteronyssinus at screening 1 or within the past 12 months (if performed and documented at the clinical unit). 5. Subject has a serum specific IgE level (≥ 0.7 kU/L) to D. pteronyssinus at screening 1 or within the past 12 months (if performed and documented at the clinical unit). 6. Subject shows a symptomatic reaction to an HDM and has a TNSS of 6 or more at least twice during the last 3 hours within the 4 hour challenge test at screening 2. 7. Subject has a FEV1 of 80% of predicted value or greater at screening 1. If subject fails to achieve this value, the assessment may be repeated 2 additional times. 8. Subject has a body mass index (BMI) range of 18.5 to 35.0 kg/m2, inclusive and weighs at least 50 kg at screening 1. 9. A female subject is eligible to participate if she is not pregnant and at least 1 of the following conditions applies:    1. Not a woman of childbearing potential (WOCBP),    2. OR    3. WOCBP who agrees to follow the contraceptive guidance, starting at screening 1 and throughout the initial safety follow-up period 10. Female subject must agree not to breastfeed starting at screening 1 and throughout the initial safety follow-up period. 11. Female subject must not donate ova starting at screening 1 and throughout the initial safety follow-up period. 12. A male subject with female partner(s) of childbearing potential must agree to use contraception until after completion of the initial safety follow-up period. 13. A male subject must not donate sperm until after completion of the initial safety follow-up period. 14. Male subject with a pregnant or breastfeeding partner(s) must agree to remain abstinent or use a condom for the duration of the pregnancy or time partner(s) is(are) breastfeeding until after completion of the initial safety follow-up period. 15. Subject agrees not to participate in another interventional study while receiving study drug in present study and until after completion of the initial safety follow-up period. |
| --- | --- |
| **Exclusion Criteria** | Subject will be excluded from participation if any of the following apply:   1. Subject with concomitant allergies to seasonal aeroallergens which are anticipated to be or become active (i.e., grass, trees, weeds, rye; defined as IgE ≥ 3.5 kU/L [IgE samples drawn within 12 months prior to screening 1 can be used to assess criteria as long as they are performed and documented at the clinical unit]; OR symptomatic to aeroallergens within the past 2 years or within the past 2 allergy seasons; OR both) through the completion of the week 18 daily symptom diary in the respective season. 2. Subject with a concomitant allergy to an animal dander who has exposure on a regular basis to the respective animal dander. 3. Subject has received immunosuppressive treatment within 3 months prior to screening 1. 4. Subject has received previous immunotherapy treatment with any HDM allergen within 3 years prior to screening 1. 5. Subject is receiving ongoing treatment with any specific immunotherapy for other allergies or plans to receive during the course of the primary study period. 6. Subject who has used systemic (or inhaled) steroid, mast cell stabilizing drug, Th2 cytokine inhibitor, thromboxane A2 synthesis inhibitor, thromboxane A2 receptor antagonist, β-blocker, α-adrenergic blockers, ergot alkaloids, angiotensin-converting enzyme inhibitors and/or angiotensin-receptor blockers within 2 months prior to first study drug administration. 7. Subject who has used biologics that are immune modulators (including anti-TNF- α antibody and anti-IgE monoclonal antibody) within 3 months prior to first study drug administration. 8. Subject who has received or is planning to receive vaccination of a live vaccine within 28 days prior to the first administration of the study drug and/or subject who has received or is planning to receive vaccination of an inactive vaccine/toxoid within 7 days prior to the first administration of the study drug during the primary study period. 9. Subject with mild to severe asthma receiving therapy consistent with the Global Initiative for Asthma treatment steps 2, 3, 4 and 5 (Global Initiative for Asthma, Global Strategy for Asthma Management and Prevention 2018: www.ginasthma.org). 10. Subject has a nasal condition that could confound the efficacy or safety assessments (e.g., nasal polyps). 11. Subject who has history of allergic reactions such as anaphylactic shock, exanthema generalized, angioedema or hypotension caused by HDM and/or any medical products (including vaccine) in the past. 12. Subject who has immune disorders (including autoimmune disease) and/or diseases requiring immunosuppressive drugs. 13. Subject who was diagnosed with immunodeficiency in the past. 14. Subject who is unable to discontinue antihistamines within 3 days or 5 half-lives (whichever duration is longer) prior to SPT and chamber. 15. Subject has received investigational study drug within 28 days or 5 half-lives, whichever is longer, prior to screening 1. 16. Subject has any condition which, in the investigator’s opinion, makes the subject unsuitable for study participation. 17. Female subject who has been pregnant within 6 months prior to screening assessment or breastfeeding at screening 1. 18. Subject has a known or suspected hypersensitivity to ASP2390, or any components of the formulation used. 19. Subject has a known or suspected intolerance to lactose and/or milk products. 20. Subject has any of the liver function tests (LFTs) (aspartate aminotransferase [AST], alanine aminotransferase [ALT], alkaline phosphatase [ALP], gamma-glutamyl transferase and total bilirubin [TBL]) ≥ 1.5 times the upper limit of normal (ULN) at screening 1. In such a case, the assessment may be repeated once. 21. Subject has severe or poorly controlled atopic dermatitis or generalized eczema. 22. Subject has any history or evidence of any clinically significant cardiovascular, gastrointestinal, endocrinologic, hematologic, hepatic, immunologic, metabolic, urologic, pulmonary, neurologic, dermatologic, psychiatric, renal and/or other major disease or malignancy, as judged by the investigator. 23. Subject has/had febrile illness or symptomatic, viral, bacterial (including upper respiratory infection), or fungal (noncutaneous) infection within 1 week prior to screening 1. 24. Subject has any clinically significant abnormality following the investigator’s review of the physical examination, ECG and protocol-defined clinical laboratory tests at screening 1. 25. Subject has a mean corrected QT interval using Fridericia’s formula (QTcF) of > 450 msec (for male subjects) and > 470 msec (for female subjects) at screening 1. If the mean QTcF exceeds the limits above, 1 additional triplicate ECG can be taken. 26. Subject has used any prescribed or nonprescribed drugs (including vitamins, natural and herbal remedies, e.g., St. John’s Wort) in the 2 weeks prior to first study drug administration, except for occasional use of paracetamol (up to 2 g/day), topical dermatological products (excluding corticosteroid products), hormonal contraceptives or hormone replacement therapy (HRT), beta-2-agonist for treatment of asthma (salbutamol), rescue medications for rhinitis (Desloratadine [oral] and Azelastine [nasal]) and for conjunctivitis (Desloratadine [oral] and Azelastine [ocular]), or the COVID-19 vaccine within 7 days prior to the first study drug administration. 27. Subject has a history of smoking more than 10 cigarettes (or equivalent amount of tobacco) per day within 3 months prior to screening 1. 28. Subject has a history of drinking more than 21 units of alcohol per week (1 unit = 10 g pure alcohol = 250 mL of beer [5%] or 35 mL of spirits [35%] or 100 mL of wine [12%]) (> 14 units of alcohol for female subjects) within 3 months prior to screening 1 or the subject tests positive for alcohol or drugs of abuse (amphetamines, barbiturates, benzodiazepines, cannabinoids, cocaine and opiates) at screening 1 or 2. 29. Subject has used any drugs of abuse (amphetamines, barbiturates, benzodiazepines, cannabinoids, cocaine and opiates) within 3 months prior to screening 1. 30. Subject has had significant blood loss, donated 1 unit (450 mL) of blood or more, or received a transfusion of any blood or blood products within 60 days or donated plasma within 7 days prior to screening 1. 31. Subject has a positive serology test for hepatitis B surface antigen (HBsAg), hepatitis B core (HBc) antibodies, hepatitis C virus (HCV) antibodies or human immunodeficiency virus (HIV) antibody at screening 1. 32. Subject is a vulnerable subject (e.g., subject kept in detention). 33. Subject is an employee of Astellas or the CRO. 34. Subject has a positive result for SARS-CoV-2 PCR test prior to the chamber challenge at screening 2 (repeat virus testing performed 4 to 7 days apart with the second PCR test to be performed a maximum of 2 days prior to the chamber challenge at screening 2) or prior to randomization on day 1. 35. Subject has clinical signs and symptoms consistent with COVID-19 infection, e.g., fever, dry cough, dyspnea, sore throat, fatigue, muscle or body aches and gastrointestinal symptoms or confirmed infection by appropriate SARS-CoV-2 PCR test within the 4 weeks prior to screening 1. |

Supplementary Table E2: Characteristics of the Participants at Baseline.

BMI: body mass index (weight [kg]/height^2^ [m^2^]); Der p: Dermatophagoides pteronyssinus allergen; Der f: Dermatophagoides farinae allergen; IgE: immunoglobulin E

| **Baseline Variable** | **Category** | **1 mg ASP2390 (N=7)** | **4 mg ASP2390 (N=13)** | **Placebo (N=8)** | **Total (N=28)** |
| --- | --- | --- | --- | --- | --- |
| **Sex** | Male/Female (%) | 6/1 (85.7/14.3) | 10/3 (76.9) | 7/1 (87.5/12.5) | 23/5 (82.1/17.9) |
| **Race** | White/Asian (%) | 6/1 (85.7/14.3) | 13/0 (100.0/0) | 8/0 (100.0/0) | 27/1 (96.4/3.6) |
| **Age (Years)** | Mean (SD) | 26.7 (4.8) | 26.1 (5.4) | 28.3 (4.5) | 26.9 (4.9) |
| **Weight (kg)** | Mean (SD) | 81.4 (10.0) | 75.9 (13.3) | 75.4 (13.9) | 77.2 (12.5) |
| **Height (cm)** | Mean (SD) | 177.6 (8.7) | 175.7 (8.0) | 179.0 (5.5) | 177.1 (7.4) |
| **BMI (kg/m^2^)** | Mean (SD) | 25.8 (1.8) | 24.6 (3.8) | 23.4 (3.5) | 24.5 (3.3) |
| **Der p-specific IgE (kU/L)** | Mean (SD) | 7.993 (8.370) | 10.770 (9.886) | 12.254 (11.475) | 10.500 (9.783) |
| **Der f-specific IgE (kU/L)** | Mean (SD) | 8.294 (6.887) | 17.458 (20.547) | 16.070 (11.935) | 14.770 (15.810) |
| **Skin Prick Test (mm)** | Mean (SD) | 8.7 (3.0) | 10.1 (4.3) | 8.3 (2.8) | 9.2 (3.6) |

Supplementary Table E3: Incidence of Treatment-emergent Adverse Events.

Number of participants (n) and percent of participants (%) are shown. Sorting order: ascending order by System Organ Class Code and descending by the number of participants of ASP2390 Total group by Preferred Term. In case of ties, ascending order by Preferred Term Code is applied.

| **MedDRA v23.0 SOC** Preferred Term | **1 mg ASP2390 (N=7) n (%)** | **4 mg ASP2390 (N=13) n (%)** | **Total ASP2390 (N=20) n (%)** | **Placebo (N=8) n (%)** |
| --- | --- | --- | --- | --- |
| **Overall** | **7 (100.0)** | **13 (100.0)** | **20 (100.0)** | **7 (87.5)** |
| **Cardiac disorders** | **0** | **1 (7.7)** | **1 (5.0)** | **1 (12.5)** |
| Palpitations | 0 | 1 (7.7) | 1 (5.0) | 0 |
| Bundle branch block right | 0 | 0 | 0 | 1 (12.5) |
| **Ear and labyrinth disorders** | **1 (14.3)** | **2 (15.4)** | **3 (15.0)** | **0** |
| Ear pain | 0 | 2 (15.4) | 2 (10.0) | 0 |
| Vertigo | 1 (14.3) | 0 | 1 (5.0) | 0 |
| **Eye disorders** | **1 (14.3)** | **0** | **1 (5.0)** | **0** |
| Eye pruritus | 1 (14.3) | 0 | 1 (5.0) | 0 |
| **Gastrointestinal disorders** | **2 (28.6)** | **2 (15.4)** | **4 (20.0)** | **2 (25.0)** |
| Diarrhoea | 2 (28.6) | 0 | 2 (10.0) | 2 (25.0) |
| Abdominal pain upper | 1 (14.3) | 0 | 1 (5.0) | 0 |
| Aphthous ulcer | 0 | 1 (7.7) | 1 (5.0) | 0 |
| Faeces soft | 0 | 1 (7.7) | 1 (5.0) | 0 |
| Food poisoning | 0 | 1 (7.7) | 1 (5.0) | 0 |
| Gingival pain | 0 | 1 (7.7) | 1 (5.0) | 0 |
| Teething | 0 | 1 (7.7) | 1 (5.0) | 0 |
| Dyspepsia | 0 | 0 | 0 | 1 (12.5) |
| **General disorders and administration site conditions** | **5 (71.4)** | **6 (46.2)** | **11 (55.0)** | **4 (50.0)** |
| Fatigue | 1 (14.3) | 2 (15.4) | 3 (15.0) | 3 (37.5) |
| Injection site pruritus | 1 (14.3) | 1 (7.7) | 2 (10.0) | 0 |
| Pyrexia | 1 (14.3) | 1 (7.7) | 2 (10.0) | 0 |
| Vaccination site pain | 1 (14.3) | 1 (7.7) | 2 (10.0) | 1 (12.5) |
| Chills | 0 | 1 (7.7) | 1 (5.0) | 0 |
| Puncture site erythema | 1 (14.3) | 0 | 1 (5.0) | 0 |
| Tenderness | 0 | 1 (7.7) | 1 (5.0) | 0 |
| Vaccination site discolouration | 0 | 1 (7.7) | 1 (5.0) | 0 |
| Chest discomfort | 0 | 0 | 0 | 1 (12.5) |
| Feeling hot | 0 | 0 | 0 | 1 (12.5) |
| **Immune system disorders** | **0** | **1 (7.7)** | **1 (5.0)** | **0** |
| Allergy to animal | 0 | 1 (7.7) | 1 (5.0) | 0 |
| **Infections and infestations** | **4 (57.1)** | **10 (76.9)** | **14 (70.0)** | **3 (37.5)** |
| Nasopharyngitis | 2 (28.6) | 8 (61.5) | 10 (50.0) | 1 (12.5) |
| COVID-19 | 0 | 8 (61.5) | 8 (40.0) | 3 (37.5) |
| Upper respiratory tract infection | 0 | 3 (23.1) | 3 (15.0) | 0 |
| Asymptomatic bacteriuria | 1 (14.3) | 0 | 1 (5.0) | 0 |
| Conjunctivitis | 1 (14.3) | 0 | 1 (5.0) | 0 |
| Gastroenteritis | 0 | 1 (7.7) | 1 (5.0) | 0 |
| Oral herpes | 1 (14.3) | 0 | 1 (5.0) | 0 |
| Influenza | 0 | 0 | 0 | 1 (12.5) |
| Urinary tract infection | 0 | 0 | 0 | 1 (12.5) |
| **Injury, poisoning and procedural complications** | **2 (28.6)** | **3 (23.1)** | **5 (25.0)** | **1 (12.5)** |
| Ligament rupture | 1 (14.3) | 1 (7.7) | 2 (10.0) | 0 |
| Ankle fracture | 0 | 1 (7.7) | 1 (5.0) | 0 |
| Arthropod sting | 1 (14.3) | 0 | 1 (5.0) | 0 |
| Bone contusion | 0 | 1 (7.7) | 1 (5.0) | 0 |
| Meniscus injury | 0 | 1 (7.7) | 1 (5.0) | 0 |
| Procedural pain | 0 | 1 (7.7) | 1 (5.0) | 0 |
| Arthropod bite | 0 | 0 | 0 | 1 (12.5) |
| **Metabolism and nutrition disorders** | **0** | **1 (7.7)** | **1 (5.0)** | **0** |
| Food intolerance | 0 | 1 (7.7) | 1 (5.0) | 0 |
| **Musculoskeletal and connective tissue disorders** | **3 (42.9)** | **2 (15.4)** | **5 (25.0)** | **1 (12.5)** |
| Back pain | 2 (28.6) | 1 (7.7) | 3 (15.0) | 0 |
| Arthralgia | 1 (14.3) | 0 | 1 (5.0) | 0 |
| Muscular weakness | 1 (14.3) | 0 | 1 (5.0) | 0 |
| Myalgia | 0 | 1 (7.7) | 1 (5.0) | 1 (12.5) |
| Pain in extremity | 0 | 1 (7.7) | 1 (5.0) | 0 |
| Tenosynovitis | 1 (14.3) | 0 | 1 (5.0) | 0 |
| **Nervous system disorders** | **5 (71.4)** | **4 (30.8)** | **9 (45.0)** | **3 (37.5)** |
| Headache | 4 (57.1) | 4 (30.8) | 8 (40.0) | 3 (37.5) |
| Ageusia | 0 | 1 (7.7) | 1 (5.0) | 0 |
| Cubital tunnel syndrome | 0 | 1 (7.7) | 1 (5.0) | 0 |
| Dysaesthesia | 1 (14.3) | 0 | 1 (5.0) | 0 |
| Neuromuscular blockade | 1 (14.3) | 0 | 1 (5.0) | 0 |
| Sciatica | 1 (14.3) | 0 | 1 (5.0) | 0 |
| **Psychiatric disorders** | **0** | **1 (7.7)** | **1 (5.0)** | **0** |
| Panic attack | 0 | 1 (7.7) | 1 (5.0) | 0 |
| **Reproductive system and breast disorders** | **0** | **3 (23.1)** | **3 (15.0)** | **0** |
| Dysmenorrhoea | 0 | 2 (15.4) | 2 (10.0) | 0 |
| Premenstrual headache | 0 | 1 (7.7) | 1 (5.0) | 0 |
| **Respiratory, thoracic and mediastinal disorders** | **3 (42.9)** | **5 (38.5)** | **8 (40.0)** | **2 (25.0)** |
| Cough | 0 | 2 (15.4) | 2 (10.0) | 0 |
| Nasal congestion | 2 (28.6) | 0 | 2 (10.0) | 0 |
| Oropharyngeal pain | 1 (14.3) | 1 (7.7) | 2 (10.0) | 0 |
| Dyspnoea | 0 | 1 (7.7) | 1 (5.0) | 0 |
| Rhinitis allergic | 0 | 1 (7.7) | 1 (5.0) | 1 (12.5) |
| Sneezing | 1 (14.3) | 0 | 1 (5.0) | 1 (12.5) |
| **Skin and subcutaneous tissue disorders** | **3 (42.9)** | **2 (15.4)** | **5 (25.0)** | **1 (12.5)** |
| Dermatitis atopic | 1 (14.3) | 0 | 1 (5.0) | 0 |
| Eczema | 0 | 1 (7.7) | 1 (5.0) | 0 |
| Pruritus allergic | 1 (14.3) | 0 | 1 (5.0) | 0 |
| Rash | 0 | 1 (7.7) | 1 (5.0) | 0 |
| Skin induration | 1 (14.3) | 0 | 1 (5.0) | 0 |
| Urticaria | 0 | 0 | 0 | 1 (12.5) |
